# 5-Benzoyl-4-hydr­oxy-6-(4-nitro­phen­yl)-4-trifluoro­meth­yl-3,4,5,6-tetrahydro­pyrimidin-2(1*H*)-one monohydrate

**DOI:** 10.1107/S160053680804124X

**Published:** 2008-12-10

**Authors:** Feng-Ling Yang, Jing Zhang, Chang-Sheng Yao

**Affiliations:** a College of Chemistry and Chemical Engineering, Xuchang University, Xuchang, Henan Province 461000, People’s Republic of China; b Luohe Medical College, Luohe, Henan Province 462002, People’s Republic of China; c School of Chemistry and Chemical Engineering, Xuzhou Normal University, Xuzhou 221116, People’s Republic of China; d Key Laboratory of Biotechnology for Medicinal Plants, Xuzhou Normal University, Xuzhou 221116, People’s Republic of China

## Abstract

The asymmetric unit of the title compound, C_18_H_14_F_3_N_3_O_5_·H_2_O, contains two independent formula units. The two heterocyclic mol­ecules differ in the orientations of the benzoyl­phenyl group with respect to the tetra­hydro­pyrimidine ring [C—C—C—C torsion angles of 64.5 (3) and 67.1 (3)°]. In both mol­ecules the pyrimidine ring adopts a half-chair conformation. The mol­ecules are linked into a two-dimensional network parallel to (001) by N—H⋯O and O—H⋯O hydrogen bonds.

## Related literature

For the bioactivity of dihydro­pyrimidines, see: Brier *et al.* (2004 ▶); Cochran *et al.* (2005 ▶); Moran *et al.* (2007 ▶); Zorkun *et al.* (2006 ▶). For the bioactivity of organofluorine compounds, see: Hermann *et al.* (2003 ▶); Ulrich (2004 ▶).
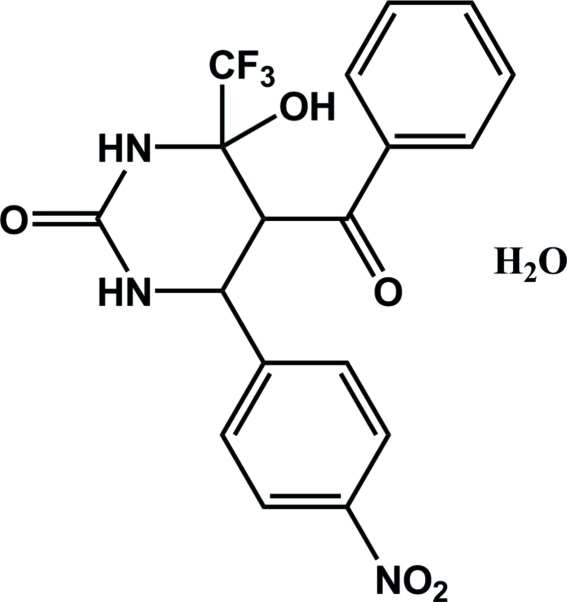


## Experimental

### 

#### Crystal data

C_18_H_14_F_3_N_3_O_5_·H_2_O
*M* *_r_* = 427.34
Orthorhombic, 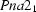

*a* = 14.389 (3) Å
*b* = 9.0391 (18) Å
*c* = 28.141 (6) Å
*V* = 3660.1 (13) Å^3^
*Z* = 8
Mo *K*α radiation
μ = 0.14 mm^−1^
*T* = 113 (2) K
0.32 × 0.22 × 0.20 mm

#### Data collection

Rigaku Saturn diffractometer
Absorption correction: multi-scan (*CrystalClear*, Rigaku/MSC, 2002 ▶) *T*_min_ = 0.958, *T*_max_ = 0.973
28524 measured reflections
4437 independent reflections
4222 reflections with *I* > 2σ(*I*)
*R*_int_ = 0.059

#### Refinement

*R*[*F* ^2^ > 2σ(*F* ^2^)] = 0.042
*wR*(*F* ^2^) = 0.107
*S* = 1.06
4437 reflections
575 parameters
2 restraints
H atoms treated by a mixture of independent and constrained refinement
Δρ_max_ = 0.25 e Å^−3^
Δρ_min_ = −0.31 e Å^−3^

### 

Data collection: *CrystalClear* (Rigaku/MSC, 2002 ▶); cell refinement: *CrystalClear*; data reduction: *CrystalClear*; program(s) used to solve structure: *SHELXS97* (Sheldrick, 2008 ▶); program(s) used to refine structure: *SHELXL97* (Sheldrick, 2008 ▶); molecular graphics: *SHELXTL* (Sheldrick, 2008 ▶); software used to prepare material for publication: *SHELXTL*.

## Supplementary Material

Crystal structure: contains datablocks I, global. DOI: 10.1107/S160053680804124X/ci2728sup1.cif

Structure factors: contains datablocks I. DOI: 10.1107/S160053680804124X/ci2728Isup2.hkl

Additional supplementary materials:  crystallographic information; 3D view; checkCIF report

## Figures and Tables

**Table 1:** Hydrogen-bond geometry (Å, °)

*D*—H⋯*A*	*D*—H	H⋯*A*	*D*⋯*A*	*D*—H⋯*A*
N2—H2*A*⋯O11	0.92 (4)	2.11 (4)	2.903 (3)	144 (3)
N1—H1⋯O11^i^	0.95 (3)	2.08 (4)	3.011 (3)	167 (3)
N5—H5⋯O12^ii^	0.89 (4)	2.05 (4)	2.907 (3)	163 (3)
N4—H4⋯O12^iii^	0.85 (4)	2.17 (4)	3.019 (3)	176 (3)
O5—H5*A*⋯O6^iv^	0.89 (4)	1.78 (4)	2.678 (3)	178 (4)
O10—H10*A*⋯O1^v^	0.85 (2)	1.82 (2)	2.672 (3)	178 (4)
O12—H12*A*⋯O5	0.85 (4)	2.13 (4)	2.817 (3)	137 (3)
O12—H12*A*⋯O2	0.85 (4)	2.32 (4)	3.026 (3)	140 (3)
O11—H11*A*⋯O10^vi^	0.82 (4)	2.22 (4)	2.815 (3)	129 (3)
O11—H11*A*⋯O7^vi^	0.82 (4)	2.27 (4)	3.009 (3)	150 (3)
O11—H11*B*⋯O6^vii^	0.83 (4)	2.05 (4)	2.853 (3)	163 (4)
O12—H12*B*⋯O1^viii^	0.82 (4)	2.12 (4)	2.840 (3)	147 (3)

Symmetry codes: (i) 
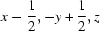
; (ii) 
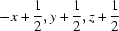
; (iii) 
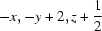
; (iv) 
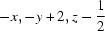
; (v) 
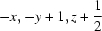
; (vi) 
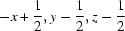
; (vii) 
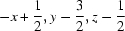
; (viii) 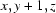.

**Fig. 1.**
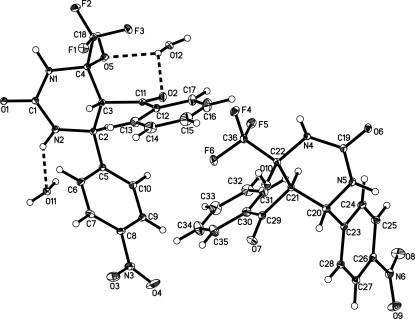
The asymmetric unit of the title compound, showing 30% probability displacement ellipsoids and the atom-numbering scheme. Hydrogen bonds are shown as dashed lines.

**Fig. 2.**
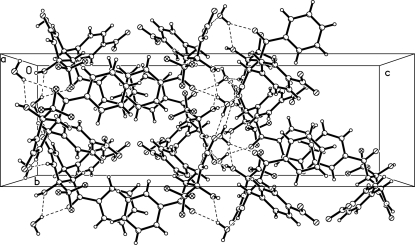
The packing diagram of the title compound. Intermolecular hydrogen bonds are shown as dashed lines.

## supplementary crystallographic information

### Comment

Dihydropyrimidine (DHPM) derivatives can be used as potential calcium channel blockers (Zorkun *et al.*,  2006), inhibitors of mitotic kinesin Eg5 for treating cancer (Cochran *et al.*,  2005; Brier *et al.*, 2004) and as TRPA1 modulators for treating pain (Moran *et al.*,  2007). Besides, compounds that contain fluorine have special bioactivity, for example, flumioxazin is a widely used herbicide (Hermann *et al.*,  2003; Ulrich,2004). This led us to pay much attention to the synthesis and bioactivity of these important fused perfluoroalkylated heterocyclic compounds. During the synthesis of DHPM derivatives, the title compound, an intermediate, was isolated and confirmed by X-ray diffraction to elucidate the reaction mechanism. We report here the crystal structure of the title compound.

The asymmetric unit of the title compound (Fig.1) contains two independent molecules which differ in the orientations of benzoylphenyl group with respect to the tetrahydropyrimidine ring. In both molecules, the pyrimidine ring adopts a half-chair conformation. The bulky substituents on the heterocyclic ring are in trans position, which must be attributed to the steric effect. The dihedral angles between C5-C10 and C12-C17 phenyl rings is 60.0 (1)° and that between C23-C28 and C30-C35 rings is 65.2 (1)°.

The crystal packing shows that the molecules are linked into a two-dimensional network parallel to the (001) by N—H···O and O—H···O hydrogen bonds (Table 1 and Fig. 2).

### Experimental

The title compound was synthesized by the reaction of 4-nitro-benzaldehyde (1 mmol), 4,4,4-trifluoro-1-phenylbutane-1,3-dione (1 mmol) and urea (1 mmol), catalyzed by 4-methylbenzenesulfonic acid, at 363 K for a certain time (monitered by TLC). After cooling, the reaction mixture was washed with water and recrystallized from ethanol, to obtain single crystals suitable for X-ray diffraction.

### Refinement

N- and O-bound H atoms were located in a difference map and were refined freely except that the O10—H10A distance was restrained to 0.88 (1) Å and the U_iso_ for O-bound H atoms were set at 1.5*U*_eq_(O). C-bound H atoms were placed in calculated positions (C-H = 0.95–1.00 Å) and included in the final cycles of refinement using a riding model, with *U*_iso_(H) = 1.2*U*_eq_(C). In the absence of significant anomalous scattering effects, Friedel pairs were averaged.

### Crystal data

**Table:**

C_18_H_14_F_3_N_3_O_5_·H_2_O	*F*(000) = 1760
*M**_r_* = 427.34	*D*_x_ = 1.551 Mg m^−^^3^
Orthorhombic, *P**n**a*2_1_	Mo *K*α radiation, λ = 0.71073 Å
Hall symbol: P 2c -2n	Cell parameters from 6634 reflections
*a* = 14.389 (3) Å	θ = 2.6–25.0°
*b* = 9.0391 (18) Å	µ = 0.14 mm^−^^1^
*c* = 28.141 (6) Å	*T* = 113 K
*V* = 3660.1 (13) Å^3^	Prism, colourless
*Z* = 8	0.32 × 0.22 × 0.20 mm

### Data collection

**Table:**

Rigaku Saturn diffractometer	4437 independent reflections
Radiation source: rotating anode	4222 reflections with *I* > 2σ(*I*)
confocal	*R*_int_ = 0.059
Detector resolution: 7.31 pixels mm^-1^	θ_max_ = 27.9°, θ_min_ = 2.4°
ω scans	*h* = −18→18
Absorption correction: multi-scan (*CrystalClear*, Rigaku/MSC, 2002)	*k* = −11→10
*T*_min_ = 0.958, *T*_max_ = 0.973	*l* = −36→37
28524 measured reflections	

### Refinement

**Table:**

Refinement on *F*^2^	Primary atom site location: structure-invariant direct methods
Least-squares matrix: full	Secondary atom site location: difference Fourier map
*R*[*F*^2^ > 2σ(*F*^2^)] = 0.042	Hydrogen site location: inferred from neighbouring sites
*wR*(*F*^2^) = 0.107	H atoms treated by a mixture of independent and constrained refinement
*S* = 1.06	*w* = 1/[σ^2^(*F*_o_^2^) + (0.0683*P*)^2^ + 0.2165*P*] where *P* = (*F*_o_^2^ + 2*F*_c_^2^)/3
4437 reflections	(Δ/σ)_max_ = 0.044
575 parameters	Δρ_max_ = 0.25 e Å^−^^3^
2 restraints	Δρ_min_ = −0.31 e Å^−^^3^

### Special details

**Table:**

Geometry. All esds (except the esd in the dihedral angle between two l.s. planes) are estimated using the full covariance matrix. The cell esds are taken into account individually in the estimation of esds in distances, angles and torsion angles; correlations between esds in cell parameters are only used when they are defined by crystal symmetry. An approximate (isotropic) treatment of cell esds is used for estimating esds involving l.s. planes.
Refinement. Refinement of F^2^ against ALL reflections. The weighted R-factor wR and goodness of fit S are based on F^2^, conventional R-factors R are based on F, with F set to zero for negative F^2^. The threshold expression of F^2^ > 2sigma(F^2^) is used only for calculating R-factors(gt) etc. and is not relevant to the choice of reflections for refinement. R-factors based on F^2^ are statistically about twice as large as those based on F, and R- factors based on ALL data will be even larger.

### Fractional atomic coordinates and isotropic or equivalent isotropic displacement parameters (Å^2^)

**Table:**

	*x*	*y*	*z*	*U*_iso_*/*U*_eq_	
N1	−0.09671 (16)	0.3614 (2)	0.59693 (8)	0.0172 (4)	
H1	−0.158 (2)	0.351 (3)	0.5850 (13)	0.022 (8)*	
N2	0.05590 (16)	0.2777 (3)	0.59022 (9)	0.0212 (5)	
H2A	0.088 (3)	0.198 (4)	0.5786 (14)	0.032 (10)*	
N3	0.40154 (18)	0.2937 (3)	0.73426 (9)	0.0275 (5)	
N4	0.17052 (16)	1.1349 (2)	0.98405 (8)	0.0163 (4)	
H4	0.117 (2)	1.137 (3)	0.9964 (13)	0.021 (8)*	
N5	0.32327 (15)	1.2159 (3)	0.99252 (9)	0.0205 (5)	
H5	0.364 (3)	1.277 (4)	1.0061 (14)	0.033 (9)*	
N6	0.66618 (17)	1.2226 (3)	0.84793 (9)	0.0246 (5)	
O1	−0.06318 (13)	0.1373 (2)	0.56457 (7)	0.0213 (4)	
O2	0.09085 (15)	0.7349 (2)	0.62345 (8)	0.0281 (5)	
O3	0.38832 (18)	0.2127 (3)	0.76856 (9)	0.0419 (6)	
O4	0.47761 (17)	0.3480 (3)	0.72524 (11)	0.0509 (7)	
O5	−0.04878 (14)	0.5953 (2)	0.56735 (7)	0.0196 (4)	
H5A	−0.101 (3)	0.613 (4)	0.5511 (14)	0.029*	
O6	0.20406 (14)	1.35887 (19)	1.01700 (7)	0.0207 (4)	
O7	0.36076 (15)	0.7632 (2)	0.95958 (8)	0.0262 (4)	
O8	0.64950 (18)	1.2982 (3)	0.81335 (9)	0.0431 (6)	
O9	0.74415 (16)	1.1810 (3)	0.85877 (10)	0.0427 (6)	
O10	0.21816 (13)	0.9012 (2)	1.01373 (7)	0.0205 (4)	
H10A	0.1681 (18)	0.889 (4)	1.0294 (13)	0.031*	
C1	−0.03449 (18)	0.2551 (3)	0.58280 (10)	0.0172 (5)	
C2	0.09768 (18)	0.4139 (3)	0.60829 (9)	0.0175 (5)	
H2	0.1185	0.4770	0.5811	0.021*	
C3	0.02400 (17)	0.4968 (3)	0.63747 (9)	0.0164 (5)	
H3	0.0100	0.4373	0.6666	0.020*	
C4	−0.06557 (18)	0.5103 (3)	0.60803 (9)	0.0155 (5)	
C5	0.17991 (18)	0.3796 (3)	0.64030 (9)	0.0180 (5)	
C6	0.17091 (19)	0.2707 (3)	0.67560 (11)	0.0227 (6)	
H6	0.1153	0.2144	0.6777	0.027*	
C7	0.2428 (2)	0.2452 (3)	0.70731 (10)	0.0229 (6)	
H7	0.2368	0.1733	0.7317	0.027*	
C8	0.32376 (19)	0.3271 (3)	0.70250 (10)	0.0200 (5)	
C9	0.33499 (19)	0.4337 (3)	0.66753 (10)	0.0224 (6)	
H9	0.3915	0.4873	0.6648	0.027*	
C10	0.26174 (19)	0.4602 (3)	0.63676 (10)	0.0207 (5)	
H10	0.2676	0.5342	0.6130	0.025*	
C11	0.06423 (19)	0.6473 (3)	0.65320 (10)	0.0191 (5)	
C12	0.0703 (2)	0.6817 (3)	0.70501 (11)	0.0228 (6)	
C13	0.0605 (2)	0.5753 (3)	0.74055 (11)	0.0296 (6)	
H13	0.0485	0.4751	0.7324	0.036*	
C14	0.0685 (3)	0.6161 (4)	0.78780 (13)	0.0411 (8)	
H14	0.0626	0.5434	0.8120	0.049*	
C15	0.0846 (3)	0.7598 (5)	0.79987 (15)	0.0499 (10)	
H15	0.0898	0.7863	0.8324	0.060*	
C16	0.0935 (3)	0.8662 (4)	0.76563 (17)	0.0517 (11)	
H16	0.1037	0.9663	0.7745	0.062*	
C17	0.0875 (3)	0.8281 (4)	0.71771 (14)	0.0361 (8)	
H17	0.0950	0.9016	0.6939	0.043*	
C18	−0.14246 (19)	0.5841 (3)	0.63835 (10)	0.0194 (5)	
C19	0.23177 (18)	1.2409 (3)	0.99904 (10)	0.0169 (5)	
C20	0.36507 (18)	1.0817 (3)	0.97328 (10)	0.0189 (5)	
H20	0.3873	1.0176	0.9999	0.023*	
C21	0.29152 (17)	0.9978 (3)	0.94407 (9)	0.0161 (5)	
H21	0.2781	1.0563	0.9147	0.019*	
C22	0.20117 (18)	0.9860 (3)	0.97330 (9)	0.0174 (5)	
C23	0.44638 (18)	1.1209 (3)	0.94105 (10)	0.0177 (5)	
C24	0.43558 (19)	1.2302 (3)	0.90652 (11)	0.0230 (6)	
H24	0.3790	1.2840	0.9047	0.028*	
C25	0.5063 (2)	1.2605 (3)	0.87512 (11)	0.0231 (6)	
H25	0.4986	1.3324	0.8508	0.028*	
C26	0.58888 (18)	1.1836 (3)	0.87981 (10)	0.0194 (5)	
C27	0.60266 (19)	1.0753 (3)	0.91366 (10)	0.0206 (5)	
H27	0.6603	1.0247	0.9161	0.025*	
C28	0.52921 (19)	1.0424 (3)	0.94424 (10)	0.0199 (5)	
H28	0.5358	0.9663	0.9673	0.024*	
C29	0.33101 (19)	0.8468 (3)	0.92912 (10)	0.0200 (5)	
C30	0.3331 (2)	0.8049 (3)	0.87787 (11)	0.0238 (6)	
C31	0.3250 (2)	0.9069 (3)	0.84102 (11)	0.0274 (6)	
H31	0.3171	1.0090	0.8480	0.033*	
C32	0.3284 (3)	0.8593 (4)	0.79407 (12)	0.0364 (8)	
H32	0.3237	0.9292	0.7690	0.044*	
C33	0.3385 (3)	0.7107 (4)	0.78375 (14)	0.0444 (9)	
H33	0.3397	0.6785	0.7516	0.053*	
C34	0.3470 (3)	0.6092 (4)	0.82007 (14)	0.0429 (9)	
H34	0.3544	0.5072	0.8128	0.052*	
C35	0.3447 (3)	0.6548 (3)	0.86693 (12)	0.0329 (7)	
H35	0.3509	0.5843	0.8918	0.040*	
C36	0.1248 (2)	0.9125 (3)	0.94281 (10)	0.0210 (5)	
F1	−0.14776 (12)	0.52363 (19)	0.68178 (6)	0.0286 (4)	
F2	−0.22576 (11)	0.57373 (19)	0.61866 (6)	0.0274 (4)	
F3	−0.12376 (13)	0.72802 (19)	0.64456 (7)	0.0324 (4)	
F4	0.04104 (12)	0.92471 (19)	0.96255 (6)	0.0280 (4)	
F5	0.12020 (12)	0.97358 (19)	0.89937 (6)	0.0278 (4)	
F6	0.14278 (13)	0.76842 (18)	0.93679 (8)	0.0322 (4)	
O11	0.21603 (13)	0.1296 (2)	0.54929 (8)	0.0212 (4)	
H11A	0.215 (3)	0.183 (4)	0.5257 (15)	0.032*	
H11B	0.228 (3)	0.046 (4)	0.5390 (14)	0.032*	
O12	0.01675 (13)	0.8681 (2)	0.53237 (8)	0.0213 (4)	
H12A	0.012 (2)	0.807 (4)	0.5554 (15)	0.032*	
H12B	0.015 (3)	0.948 (4)	0.5459 (14)	0.032*	

### Atomic displacement parameters (Å^2^)

**Table:**

	*U*^11^	*U*^22^	*U*^33^	*U*^12^	*U*^13^	*U*^23^
N1	0.0173 (11)	0.0159 (10)	0.0185 (10)	−0.0003 (8)	−0.0023 (9)	−0.0037 (8)
N2	0.0182 (11)	0.0221 (12)	0.0233 (12)	0.0018 (9)	−0.0025 (9)	−0.0082 (9)
N3	0.0243 (12)	0.0319 (13)	0.0263 (13)	0.0078 (10)	−0.0046 (10)	−0.0066 (11)
N4	0.0139 (11)	0.0149 (10)	0.0200 (11)	0.0003 (8)	0.0022 (9)	−0.0035 (8)
N5	0.0146 (10)	0.0204 (11)	0.0265 (13)	−0.0035 (8)	0.0029 (9)	−0.0085 (10)
N6	0.0225 (12)	0.0256 (11)	0.0257 (13)	−0.0043 (9)	0.0055 (10)	−0.0005 (10)
O1	0.0200 (9)	0.0186 (9)	0.0254 (10)	0.0006 (7)	−0.0051 (8)	−0.0064 (7)
O2	0.0314 (11)	0.0223 (9)	0.0306 (12)	−0.0066 (8)	−0.0053 (9)	0.0047 (8)
O3	0.0397 (14)	0.0552 (15)	0.0309 (13)	0.0083 (11)	−0.0100 (10)	0.0120 (12)
O4	0.0212 (12)	0.0774 (19)	0.0542 (17)	−0.0049 (12)	−0.0122 (11)	0.0189 (15)
O5	0.0198 (10)	0.0227 (9)	0.0162 (9)	−0.0035 (7)	−0.0041 (7)	0.0070 (7)
O6	0.0205 (10)	0.0172 (9)	0.0246 (10)	−0.0014 (7)	0.0049 (8)	−0.0074 (7)
O7	0.0313 (11)	0.0257 (10)	0.0217 (11)	0.0099 (9)	0.0031 (8)	0.0039 (8)
O8	0.0383 (14)	0.0547 (15)	0.0363 (14)	0.0066 (11)	0.0154 (11)	0.0214 (13)
O9	0.0190 (11)	0.0639 (16)	0.0453 (14)	0.0005 (11)	0.0045 (10)	0.0152 (12)
O10	0.0176 (10)	0.0233 (9)	0.0206 (9)	0.0010 (7)	0.0033 (7)	0.0070 (8)
C1	0.0186 (12)	0.0169 (12)	0.0161 (13)	0.0005 (10)	−0.0019 (10)	−0.0006 (9)
C2	0.0157 (12)	0.0177 (11)	0.0191 (12)	−0.0010 (9)	0.0000 (9)	−0.0006 (9)
C3	0.0161 (12)	0.0171 (11)	0.0159 (11)	−0.0010 (9)	−0.0020 (9)	−0.0005 (9)
C4	0.0163 (12)	0.0152 (11)	0.0150 (11)	0.0005 (9)	−0.0020 (9)	0.0003 (9)
C5	0.0179 (13)	0.0175 (12)	0.0187 (12)	0.0005 (9)	−0.0014 (10)	−0.0027 (9)
C6	0.0165 (12)	0.0248 (13)	0.0267 (15)	−0.0027 (10)	−0.0009 (11)	0.0028 (11)
C7	0.0249 (14)	0.0238 (13)	0.0200 (15)	0.0072 (11)	0.0004 (11)	0.0026 (10)
C8	0.0166 (13)	0.0243 (12)	0.0192 (12)	0.0052 (10)	−0.0033 (10)	−0.0051 (10)
C9	0.0163 (12)	0.0233 (13)	0.0277 (15)	−0.0012 (10)	−0.0008 (11)	−0.0038 (11)
C10	0.0206 (13)	0.0204 (12)	0.0210 (13)	−0.0002 (10)	−0.0011 (10)	0.0003 (10)
C11	0.0172 (12)	0.0166 (12)	0.0234 (13)	−0.0005 (9)	−0.0063 (10)	−0.0005 (10)
C12	0.0225 (13)	0.0210 (13)	0.0249 (14)	0.0008 (10)	−0.0059 (11)	−0.0067 (11)
C13	0.0373 (17)	0.0302 (15)	0.0214 (14)	0.0032 (12)	−0.0072 (12)	−0.0052 (12)
C14	0.050 (2)	0.050 (2)	0.0231 (16)	0.0086 (17)	−0.0084 (15)	−0.0097 (15)
C15	0.059 (3)	0.064 (2)	0.0273 (19)	0.001 (2)	−0.0115 (17)	−0.0219 (18)
C16	0.057 (2)	0.042 (2)	0.056 (3)	−0.0058 (17)	−0.011 (2)	−0.032 (2)
C17	0.0393 (19)	0.0251 (15)	0.044 (2)	−0.0038 (13)	−0.0058 (15)	−0.0093 (14)
C18	0.0193 (13)	0.0186 (12)	0.0202 (13)	−0.0016 (10)	−0.0013 (10)	−0.0007 (10)
C19	0.0183 (12)	0.0168 (12)	0.0156 (12)	−0.0015 (10)	0.0018 (10)	−0.0011 (9)
C20	0.0171 (12)	0.0204 (12)	0.0193 (12)	0.0002 (9)	0.0039 (10)	−0.0011 (10)
C21	0.0159 (12)	0.0148 (11)	0.0174 (12)	−0.0002 (9)	0.0012 (9)	0.0012 (9)
C22	0.0192 (12)	0.0151 (11)	0.0178 (12)	−0.0001 (9)	0.0020 (9)	0.0005 (9)
C23	0.0154 (13)	0.0187 (12)	0.0191 (12)	−0.0017 (9)	0.0004 (10)	−0.0034 (10)
C24	0.0160 (12)	0.0257 (13)	0.0272 (15)	0.0000 (10)	−0.0012 (11)	0.0025 (11)
C25	0.0219 (13)	0.0239 (13)	0.0234 (15)	0.0018 (11)	−0.0005 (11)	0.0047 (11)
C26	0.0170 (13)	0.0221 (12)	0.0193 (12)	−0.0040 (9)	0.0022 (10)	−0.0016 (10)
C27	0.0193 (13)	0.0193 (12)	0.0231 (14)	0.0012 (10)	0.0014 (10)	−0.0004 (10)
C28	0.0199 (13)	0.0202 (12)	0.0196 (12)	0.0012 (10)	−0.0013 (10)	−0.0012 (10)
C29	0.0169 (13)	0.0201 (13)	0.0231 (14)	−0.0009 (10)	0.0038 (10)	−0.0012 (10)
C30	0.0224 (14)	0.0258 (14)	0.0233 (14)	0.0005 (11)	0.0076 (11)	−0.0031 (11)
C31	0.0305 (16)	0.0267 (14)	0.0249 (15)	0.0002 (12)	0.0049 (12)	−0.0012 (12)
C32	0.0425 (19)	0.0447 (19)	0.0221 (16)	0.0049 (15)	0.0058 (14)	−0.0001 (14)
C33	0.055 (2)	0.053 (2)	0.0260 (18)	0.0000 (17)	0.0102 (16)	−0.0170 (16)
C34	0.063 (3)	0.0310 (16)	0.0350 (18)	0.0015 (16)	0.0148 (17)	−0.0141 (14)
C35	0.0451 (19)	0.0243 (14)	0.0294 (17)	0.0044 (13)	0.0100 (14)	−0.0068 (12)
C36	0.0214 (14)	0.0177 (12)	0.0238 (13)	−0.0017 (10)	0.0015 (11)	−0.0021 (10)
F1	0.0313 (9)	0.0361 (9)	0.0182 (8)	0.0027 (7)	0.0060 (7)	0.0003 (7)
F2	0.0177 (8)	0.0352 (9)	0.0293 (9)	0.0046 (7)	−0.0009 (7)	−0.0078 (7)
F3	0.0316 (10)	0.0180 (8)	0.0475 (13)	0.0040 (7)	0.0041 (8)	−0.0103 (8)
F4	0.0183 (8)	0.0348 (9)	0.0310 (9)	−0.0038 (7)	0.0037 (7)	−0.0104 (8)
F5	0.0300 (9)	0.0331 (9)	0.0202 (8)	−0.0026 (7)	−0.0041 (7)	−0.0016 (7)
F6	0.0328 (10)	0.0178 (8)	0.0459 (12)	−0.0009 (7)	−0.0003 (8)	−0.0105 (8)
O11	0.0228 (10)	0.0166 (9)	0.0242 (10)	0.0021 (8)	−0.0009 (8)	0.0028 (8)
O12	0.0242 (11)	0.0171 (9)	0.0224 (10)	0.0020 (7)	0.0009 (8)	0.0011 (8)

### Geometric parameters (Å, °)

**Table:**

N1—C1	1.372 (3)	C13—H13	0.95
N1—C4	1.452 (3)	C14—C15	1.362 (6)
N1—H1	0.95 (3)	C14—H14	0.95
N2—C1	1.333 (3)	C15—C16	1.367 (7)
N2—C2	1.461 (3)	C15—H15	0.95
N2—H2A	0.92 (4)	C16—C17	1.395 (6)
N3—O4	1.226 (4)	C16—H16	0.95
N3—O3	1.226 (4)	C17—H17	0.95
N3—C8	1.464 (3)	C18—F2	1.324 (3)
N4—C19	1.368 (3)	C18—F3	1.340 (3)
N4—C22	1.449 (3)	C18—F1	1.341 (3)
N4—H4	0.85 (4)	C20—C23	1.522 (4)
N5—C19	1.348 (3)	C20—C21	1.540 (4)
N5—C20	1.458 (3)	C20—H20	1.00
N5—H5	0.89 (4)	C21—C29	1.537 (4)
N6—O8	1.213 (4)	C21—C22	1.542 (3)
N6—O9	1.222 (3)	C21—H21	1.00
N6—C26	1.472 (3)	C22—C36	1.545 (4)
O1—C1	1.252 (3)	C23—C28	1.390 (4)
O2—C11	1.214 (4)	C23—C24	1.394 (4)
O5—C4	1.400 (3)	C24—C25	1.375 (4)
O5—H5A	0.89 (4)	C24—H24	0.95
O6—C19	1.246 (3)	C25—C26	1.383 (4)
O7—C29	1.221 (3)	C25—H25	0.95
O10—C22	1.393 (3)	C26—C27	1.380 (4)
O10—H10A	0.852 (19)	C27—C28	1.395 (4)
C2—C5	1.519 (4)	C27—H27	0.95
C2—C3	1.536 (4)	C28—H28	0.95
C2—H2	1.00	C29—C30	1.491 (4)
C3—C4	1.537 (3)	C30—C31	1.393 (4)
C3—C11	1.544 (4)	C30—C35	1.401 (4)
C3—H3	1.00	C31—C32	1.390 (4)
C4—C18	1.548 (4)	C31—H31	0.95
C5—C10	1.388 (4)	C32—C33	1.382 (5)
C5—C6	1.405 (4)	C32—H32	0.95
C6—C7	1.385 (4)	C33—C34	1.379 (6)
C6—H6	0.95	C33—H33	0.95
C7—C8	1.387 (4)	C34—C35	1.382 (5)
C7—H7	0.95	C34—H34	0.95
C8—C9	1.387 (4)	C35—H35	0.95
C9—C10	1.385 (4)	C36—F4	1.331 (3)
C9—H9	0.95	C36—F6	1.338 (3)
C10—H10	0.95	C36—F5	1.343 (3)
C11—C12	1.493 (4)	O11—H11A	0.82 (4)
C12—C17	1.393 (4)	O11—H11B	0.83 (4)
C12—C13	1.395 (4)	O12—H12A	0.85 (4)
C13—C14	1.385 (4)	O12—H12B	0.82 (4)
			
C1—N1—C4	120.7 (2)	C12—C17—C16	119.6 (4)
C1—N1—H1	116 (2)	C12—C17—H17	120.2
C4—N1—H1	116.9 (19)	C16—C17—H17	120.2
C1—N2—C2	125.8 (2)	F2—C18—F3	107.8 (2)
C1—N2—H2A	108 (2)	F2—C18—F1	107.5 (2)
C2—N2—H2A	125 (2)	F3—C18—F1	106.8 (2)
O4—N3—O3	122.7 (3)	F2—C18—C4	112.7 (2)
O4—N3—C8	118.3 (3)	F3—C18—C4	110.3 (2)
O3—N3—C8	119.0 (3)	F1—C18—C4	111.5 (2)
C19—N4—C22	121.3 (2)	O6—C19—N5	120.7 (2)
C19—N4—H4	117 (2)	O6—C19—N4	121.2 (2)
C22—N4—H4	112 (2)	N5—C19—N4	118.0 (2)
C19—N5—C20	126.3 (2)	N5—C20—C23	110.1 (2)
C19—N5—H5	118 (3)	N5—C20—C21	108.9 (2)
C20—N5—H5	114 (3)	C23—C20—C21	109.0 (2)
O8—N6—O9	123.7 (3)	N5—C20—H20	109.6
O8—N6—C26	118.3 (3)	C23—C20—H20	109.6
O9—N6—C26	117.9 (3)	C21—C20—H20	109.6
C4—O5—H5A	112 (2)	C29—C21—C20	109.2 (2)
C22—O10—H10A	110 (3)	C29—C21—C22	113.3 (2)
O1—C1—N2	121.1 (2)	C20—C21—C22	109.2 (2)
O1—C1—N1	119.9 (2)	C29—C21—H21	108.3
N2—C1—N1	118.9 (2)	C20—C21—H21	108.3
N2—C2—C5	110.8 (2)	C22—C21—H21	108.3
N2—C2—C3	108.2 (2)	O10—C22—N4	113.2 (2)
C5—C2—C3	108.7 (2)	O10—C22—C21	109.0 (2)
N2—C2—H2	109.7	N4—C22—C21	107.7 (2)
C5—C2—H2	109.7	O10—C22—C36	110.0 (2)
C3—C2—H2	109.7	N4—C22—C36	107.4 (2)
C2—C3—C4	109.2 (2)	C21—C22—C36	109.5 (2)
C2—C3—C11	108.9 (2)	C28—C23—C24	120.1 (2)
C4—C3—C11	113.5 (2)	C28—C23—C20	120.1 (2)
C2—C3—H3	108.3	C24—C23—C20	119.6 (2)
C4—C3—H3	108.3	C25—C24—C23	120.4 (3)
C11—C3—H3	108.3	C25—C24—H24	119.8
O5—C4—N1	112.7 (2)	C23—C24—H24	119.8
O5—C4—C3	109.8 (2)	C24—C25—C26	118.3 (3)
N1—C4—C3	107.5 (2)	C24—C25—H25	120.8
O5—C4—C18	109.7 (2)	C26—C25—H25	120.8
N1—C4—C18	107.3 (2)	C27—C26—C25	123.1 (3)
C3—C4—C18	109.7 (2)	C27—C26—N6	118.8 (2)
C10—C5—C6	119.8 (2)	C25—C26—N6	118.1 (2)
C10—C5—C2	120.7 (2)	C26—C27—C28	117.9 (3)
C6—C5—C2	119.4 (2)	C26—C27—H27	121.1
C7—C6—C5	120.2 (3)	C28—C27—H27	121.1
C7—C6—H6	119.9	C23—C28—C27	120.1 (3)
C5—C6—H6	119.9	C23—C28—H28	120.0
C6—C7—C8	118.4 (3)	C27—C28—H28	120.0
C6—C7—H7	120.8	O7—C29—C30	121.0 (3)
C8—C7—H7	120.8	O7—C29—C21	119.2 (3)
C9—C8—C7	122.6 (2)	C30—C29—C21	119.9 (2)
C9—C8—N3	119.2 (2)	C31—C30—C35	119.2 (3)
C7—C8—N3	118.2 (3)	C31—C30—C29	123.4 (3)
C10—C9—C8	118.4 (3)	C35—C30—C29	117.5 (3)
C10—C9—H9	120.8	C32—C31—C30	120.0 (3)
C8—C9—H9	120.8	C32—C31—H31	120.0
C9—C10—C5	120.7 (3)	C30—C31—H31	120.0
C9—C10—H10	119.7	C33—C32—C31	120.3 (3)
C5—C10—H10	119.7	C33—C32—H32	119.9
O2—C11—C12	121.3 (2)	C31—C32—H32	119.9
O2—C11—C3	119.7 (3)	C34—C33—C32	120.0 (3)
C12—C11—C3	119.0 (2)	C34—C33—H33	120.0
C17—C12—C13	119.3 (3)	C32—C33—H33	120.0
C17—C12—C11	117.3 (3)	C33—C34—C35	120.4 (3)
C13—C12—C11	123.4 (2)	C33—C34—H34	119.8
C14—C13—C12	119.8 (3)	C35—C34—H34	119.8
C14—C13—H13	120.1	C34—C35—C30	120.1 (3)
C12—C13—H13	120.1	C34—C35—H35	119.9
C15—C14—C13	120.5 (4)	C30—C35—H35	120.0
C15—C14—H14	119.7	F4—C36—F6	108.0 (2)
C13—C14—H14	119.7	F4—C36—F5	107.5 (2)
C14—C15—C16	120.7 (4)	F6—C36—F5	107.1 (2)
C14—C15—H15	119.6	F4—C36—C22	112.1 (2)
C16—C15—H15	119.6	F6—C36—C22	110.6 (2)
C15—C16—C17	120.1 (3)	F5—C36—C22	111.3 (2)
C15—C16—H16	119.9	H11A—O11—H11B	105 (4)
C17—C16—H16	119.9	H12A—O12—H12B	102 (4)
			
C2—N2—C1—O1	175.8 (3)	C20—N5—C19—O6	178.1 (3)
C2—N2—C1—N1	−6.4 (4)	C20—N5—C19—N4	−3.9 (4)
C4—N1—C1—O1	−166.1 (2)	C22—N4—C19—O6	−165.8 (2)
C4—N1—C1—N2	16.1 (4)	C22—N4—C19—N5	16.1 (4)
C1—N2—C2—C5	144.1 (3)	C19—N5—C20—C23	141.1 (3)
C1—N2—C2—C3	25.0 (4)	C19—N5—C20—C21	21.7 (4)
N2—C2—C3—C4	−50.6 (3)	N5—C20—C21—C29	−172.7 (2)
C5—C2—C3—C4	−171.0 (2)	C23—C20—C21—C29	67.1 (3)
N2—C2—C3—C11	−175.1 (2)	N5—C20—C21—C22	−48.3 (3)
C5—C2—C3—C11	64.5 (3)	C23—C20—C21—C22	−168.5 (2)
C1—N1—C4—O5	78.2 (3)	C19—N4—C22—O10	76.4 (3)
C1—N1—C4—C3	−43.0 (3)	C19—N4—C22—C21	−44.1 (3)
C1—N1—C4—C18	−160.9 (2)	C19—N4—C22—C36	−162.0 (2)
C2—C3—C4—O5	−63.2 (2)	C29—C21—C22—O10	58.1 (3)
C11—C3—C4—O5	58.6 (3)	C20—C21—C22—O10	−63.9 (2)
C2—C3—C4—N1	59.8 (3)	C29—C21—C22—N4	−178.7 (2)
C11—C3—C4—N1	−178.4 (2)	C20—C21—C22—N4	59.3 (3)
C2—C3—C4—C18	176.1 (2)	C29—C21—C22—C36	−62.3 (3)
C11—C3—C4—C18	−62.1 (3)	C20—C21—C22—C36	175.7 (2)
N2—C2—C5—C10	136.7 (3)	N5—C20—C23—C28	135.7 (2)
C3—C2—C5—C10	−104.5 (3)	C21—C20—C23—C28	−104.9 (3)
N2—C2—C5—C6	−46.9 (3)	N5—C20—C23—C24	−47.9 (3)
C3—C2—C5—C6	71.9 (3)	C21—C20—C23—C24	71.5 (3)
C10—C5—C6—C7	1.0 (4)	C28—C23—C24—C25	0.5 (4)
C2—C5—C6—C7	−175.4 (3)	C20—C23—C24—C25	−175.8 (3)
C5—C6—C7—C8	−1.4 (4)	C23—C24—C25—C26	−2.2 (4)
C6—C7—C8—C9	0.5 (4)	C24—C25—C26—C27	1.8 (4)
C6—C7—C8—N3	−176.4 (3)	C24—C25—C26—N6	−176.5 (3)
O4—N3—C8—C9	−8.8 (4)	O8—N6—C26—C27	167.5 (3)
O3—N3—C8—C9	171.4 (3)	O9—N6—C26—C27	−15.0 (4)
O4—N3—C8—C7	168.2 (3)	O8—N6—C26—C25	−14.2 (4)
O3—N3—C8—C7	−11.5 (4)	O9—N6—C26—C25	163.4 (3)
C7—C8—C9—C10	0.9 (4)	C25—C26—C27—C28	0.3 (4)
N3—C8—C9—C10	177.8 (2)	N6—C26—C27—C28	178.5 (2)
C8—C9—C10—C5	−1.3 (4)	C24—C23—C28—C27	1.6 (4)
C6—C5—C10—C9	0.4 (4)	C20—C23—C28—C27	177.9 (2)
C2—C5—C10—C9	176.8 (2)	C26—C27—C28—C23	−2.0 (4)
C2—C3—C11—O2	58.5 (3)	C20—C21—C29—O7	55.1 (3)
C4—C3—C11—O2	−63.4 (3)	C22—C21—C29—O7	−66.9 (3)
C2—C3—C11—C12	−121.0 (3)	C20—C21—C29—C30	−124.5 (3)
C4—C3—C11—C12	117.1 (3)	C22—C21—C29—C30	113.6 (3)
O2—C11—C12—C17	14.1 (4)	O7—C29—C30—C31	−161.6 (3)
C3—C11—C12—C17	−166.4 (3)	C21—C29—C30—C31	17.9 (4)
O2—C11—C12—C13	−165.2 (3)	O7—C29—C30—C35	17.9 (4)
C3—C11—C12—C13	14.3 (4)	C21—C29—C30—C35	−162.6 (3)
C17—C12—C13—C14	−0.3 (5)	C35—C30—C31—C32	0.0 (5)
C11—C12—C13—C14	179.0 (3)	C29—C30—C31—C32	179.5 (3)
C12—C13—C14—C15	0.8 (6)	C30—C31—C32—C33	0.9 (5)
C13—C14—C15—C16	−0.1 (6)	C31—C32—C33—C34	−1.1 (6)
C14—C15—C16—C17	−1.0 (7)	C32—C33—C34—C35	0.5 (6)
C13—C12—C17—C16	−0.8 (5)	C33—C34—C35—C30	0.4 (6)
C11—C12—C17—C16	179.8 (3)	C31—C30—C35—C34	−0.6 (5)
C15—C16—C17—C12	1.5 (6)	C29—C30—C35—C34	179.9 (3)
O5—C4—C18—F2	71.4 (3)	O10—C22—C36—F4	72.3 (3)
N1—C4—C18—F2	−51.4 (3)	N4—C22—C36—F4	−51.3 (3)
C3—C4—C18—F2	−167.9 (2)	C21—C22—C36—F4	−167.9 (2)
O5—C4—C18—F3	−49.1 (3)	O10—C22—C36—F6	−48.3 (3)
N1—C4—C18—F3	−171.9 (2)	N4—C22—C36—F6	−171.9 (2)
C3—C4—C18—F3	71.6 (3)	C21—C22—C36—F6	71.5 (3)
O5—C4—C18—F1	−167.6 (2)	O10—C22—C36—F5	−167.2 (2)
N1—C4—C18—F1	69.7 (3)	N4—C22—C36—F5	69.2 (3)
C3—C4—C18—F1	−46.8 (3)	C21—C22—C36—F5	−47.5 (3)

### Hydrogen-bond geometry (Å, °)

**Table:**

*D*—H···*A*	*D*—H	H···*A*	*D*···*A*	*D*—H···*A*
N2—H2A···O11	0.92 (4)	2.11 (4)	2.903 (3)	144 (3)
N1—H1···O11^i^	0.95 (3)	2.08 (4)	3.011 (3)	167 (3)
N5—H5···O12^ii^	0.89 (4)	2.05 (4)	2.907 (3)	163 (3)
N4—H4···O12^iii^	0.85 (4)	2.17 (4)	3.019 (3)	176 (3)
O5—H5A···O6^iv^	0.89 (4)	1.78 (4)	2.678 (3)	178 (4)
O10—H10A···O1^v^	0.85 (2)	1.82 (2)	2.672 (3)	178 (4)
O12—H12A···O5	0.85 (4)	2.13 (4)	2.817 (3)	137 (3)
O12—H12A···O2	0.85 (4)	2.32 (4)	3.026 (3)	140 (3)
O11—H11A···O10^vi^	0.82 (4)	2.22 (4)	2.815 (3)	129 (3)
O11—H11A···O7^vi^	0.82 (4)	2.27 (4)	3.009 (3)	150 (3)
O11—H11B···O6^vii^	0.83 (4)	2.05 (4)	2.853 (3)	163 (4)
O12—H12B···O1^viii^	0.82 (4)	2.12 (4)	2.840 (3)	147 (3)

Symmetry codes: (i) *x*−1/2, −*y*+1/2, *z*; (ii) −*x*+1/2, *y*+1/2, *z*+1/2; (iii) −*x*, −*y*+2, *z*+1/2; (iv) −*x*, −*y*+2, *z*−1/2; (v) −*x*, −*y*+1, *z*+1/2; (vi) −*x*+1/2, *y*−1/2, *z*−1/2; (vii) −*x*+1/2, *y*−3/2, *z*−1/2; (viii) *x*, *y*+1, *z*.
